# Association of Asthma Risk Alleles With Acute Respiratory Tract Infections and Wheezing Illnesses in Young Children

**DOI:** 10.1093/infdis/jiad075

**Published:** 2023-03-27

**Authors:** Ville Forsström, Laura Toivonen, Kiara Homil, Matti Waris, Casper-Emil T Pedersen, Klaus Bønnelykke, Tuomas Jartti, Ville Peltola

**Affiliations:** Department of Pediatrics and Adolescent Medicine, Turku University Hospital and University of Turku, Turku, Finland; Department of Pediatrics and Adolescent Medicine, Turku University Hospital and University of Turku, Turku, Finland; Department of Pediatrics and Adolescent Medicine, Turku University Hospital and University of Turku, Turku, Finland; Virology Unit, Institute of Biomedicine, University of Turku, Turku, Finland; Copenhagen Prospective Studies on Asthma in Childhood, Herlev and Gentofte Hospital, University of Copenhagen, Copenhagen, Denmark; Copenhagen Prospective Studies on Asthma in Childhood, Herlev and Gentofte Hospital, University of Copenhagen, Copenhagen, Denmark; Department of Pediatrics and Adolescent Medicine, Turku University Hospital and University of Turku, Turku, Finland; Research Unit for Pediatrics, Dermatology, Clinical Genetics, Obstetrics and Gynecology, University of Oulu, Oulu, Finland; Department of Pediatrics and Adolescent Medicine, University of Oulu, Oulu, Finland; Department of Pediatrics and Adolescent Medicine, Turku University Hospital and University of Turku, Turku, Finland

**Keywords:** asthma, acute respiratory tract infection, genetic risk, wheezing

## Abstract

**Background:**

Genome-wide association studies have identified several risk alleles for early childhood asthma, particularly in the 17q21 locus and in the cadherin-related family member 3 (*CDHR3*) gene. Contribution of these alleles to the risk of acute respiratory tract infections (ARI) in early childhood is unclear.

**Methods:**

We analyzed data from the STEPS birth-cohort study of unselected children and the VINKU and VINKU2 studies on children with severe wheezing illness. Genome-wide genotyping was performed on 1011 children. We analyzed the association between 11 preselected asthma risk alleles and the risk of ARIs and wheezing illnesses of various viral etiologies.

**Results:**

The asthma risk alleles in *CDHR3*, *GSDMA*, and *GSDMB* were associated with an increased rate of ARIs (for *CDHR3*, incidence rate ratio [IRR], 1.06; 95% confidence interval [CI], 1.01–1.12; *P* = .02), and risk allele in *CDHR3* gene with rhinovirus infections (IRR, 1.10; 95% CI, 1.01–1.20, *P* = .03). Asthma risk alleles in *GSDMA*, *GSDMB*, *IKZF3*, *ZPBP2*, and *ORMDL3* genes were associated with wheezing illnesses in early childhood, especially rhinovirus-positive wheezing illnesses.

**Conclusions:**

Asthma risk alleles were associated with an increased rate of ARIs and an increased risk of viral wheezing illnesses. Nonwheezing and wheezing ARIs and asthma may have shared genetic risk factors.

**Clinical Trials Registration**. NCT00494624 and NCT00731575.

## Teaser

Previously identified asthma risk alleles were associated with an increased rate of acute respiratory infections in early childhood. Risk allele *CDHR3*, coding for rhinovirus species C receptor, was associated with an increased rate of rhinovirus infections.

Asthma is the most prevalent chronic disease in children globally [1]. Risk factors for childhood asthma include host risk factors such as atopy and parental asthma and environmental risk factors such as exposure to tobacco smoke and reduced microbial exposure [2–5]. Development of high-throughput genetic sequencing methods has advanced the study of genetic risk factors for asthma.

Genome wide association studies (GWAS) of asthma and subsequent confirmation studies have identified several risk alleles for early childhood asthma, particularly in cadherin-related family member 3 (*CDHR3*) gene and in the 17q21 locus [6–14]. *CDHR3* encodes a CDHR3 protein which is a transmembrane protein mainly expressed in the epithelium of the airways and has been identified as a receptor for species C rhinoviruses (RVs) [15]. A single-nucleotide polymorphism (SNP) in *CDHR3* (rs6967330) has been reported to increase the surface expression of CDHR3 and associate with severe exacerbations of asthma in children [9, 16]. In the 17q21 locus, SNPs in multiple genes that are in strong linkage disequilibrium (LD) have been associated with an increased risk and varying severity of asthma, for example, SNPs in genes gasdermin A (*GSDMA*), gasdermin B (*GSDMB*), Ikaros family zinc finger 3 (*IKZF3*), zona pellucida binding protein 2 (*ZPBP2*), and ORM1-like protein 3 (*ORMDL3*) [6–14]. The effects of these alleles on asthma risk are modest and the mechanisms of the associations not yet fully understood, probably because of the multifactorial nature of asthma pathogenesis and various asthma phenotypes [14]. Many of the identified asthma risk alleles also contribute to childhood wheezing illnesses [10, 11, 17, 18], which are known to predict the risk of later development of childhood asthma. Interaction between RV wheezing illnesses in childhood and 17q21 variants further increases the asthma risk [17]. However, the majority of viral respiratory tract infections do not manifest with wheezing, and little is known about the associations between these risk alleles and the rate of all acute respiratory tract infections (ARIs). Increased number of ARIs during early childhood is a risk factor for later asthma, which suggests the possibility of shared genetic background [19].

To address this knowledge gap, we first investigated the associations between known asthma risk alleles and the rate of viral ARIs in early childhood in the Steps to the Healthy Development and Well-being of Children (STEPS) prospective birth-cohort study. Our primary aim was to investigate whether the known asthma risk alleles are associated with an increased rate of all viral ARIs. Second, we further investigated whether the known asthma risk alleles are associated with an increased rate of nonwheezing and wheezing ARIs, respectively. Further, we analyzed the risk of severe wheezing illnesses using children in the VINKU and VINKU2 trials as cases and STEPS study children who did not develop wheezing illness as controls.

## METHODS

### Study Design and Subjects

This study uses data from 3 studies conducted in the Hospital District of Southwest Finland, the STEPS study and the VINKU and VINKU2 studies. The STEPS study is a prospective birth-cohort study where 923 children born between 2008 and 2010 were enrolled at birth in an intensive follow-up for ARIs, as earlier described [19–21]. No selection criteria other than language (Finnish or Swedish speaking family) were applied in recruiting the participants in the STEPS study. The children were followed for ARIs with parent-reported daily symptom diaries and study clinic visits from birth until age 24 months. Parents were encouraged to take the children to the study clinic if they developed symptoms of ARI. A nasal swab sample was collected during ARIs at the study clinic by a study physician or at home by parents who then sent the swabs to the study clinic as earlier described [20]. Data on outpatient and emergency department visits at hospitals and hospitalizations from birth to age 24 months were retrieved from medical records of the Hospital District of Southwest Finland.

In the VINKU study (NCT00494624), as part of an efficacy trial of oral prednisolone on wheezing requiring hospitalization, 131 children aged 3–35 months were enrolled for the first acute wheezing episode between September 2000 and May 2002 [22, 23]. Exclusion criteria were any other chronic disease than allergy and asthma, varicella or recent exposure to varicella, systemic glucocorticoid treatment in the past 4 weeks prior to study, or severe disease defined as oxygen saturation below 92% despite additional oxygen and frequent salbutamol inhalations or need of treatment in intensive care unit. A nasal swab was taken at study entry.

The VINKU2 study (NCT00731575) enrolled 124 children born at gestational week 36 or later who were treated at hospital for first acute wheezing episode at the age of 3–23 months between June 2007 and October 2009, as earlier described [24, 25]. In the VINKU2 study, long-term effectiveness of short course of oral prednisolone during the first RV induced severe wheezing episode was evaluated in children in a randomized controlled trial. Main exclusion criteria were chronic nonatopic illness, previous systemic or inhaled corticosteroid treatment, participation in another trial and need for intensive care unit treatment. Nasal swab was taken at study entry.

The STEPS study and the VINKU and VINKU2 studies were approved by the Ethics Committee of the Hospital District of Southwest Finland. Parents of participating children gave their written, informed consent.

### Respiratory Virus Detection and Genotyping

In the STEPS study, nucleic acids were extracted from the nasal swabs collected during ARIs using NucliSense easyMag (BioMerieux) or MagnaPure 96 (Roche). RV, respiratory syncytial virus (RSV), enteroviruses, and influenza A and B viruses were analyzed using polymerase chain reaction (PCR) [21]. Genome-wide genotyping was performed from blood samples collected at the age of 2 months.

In the VINKU study, nasal swabs taken at study entry were analyzed using PCR to detect RV, RSV, enteroviruses, coronaviruses, and human metapneumovirus [22, 23]. Antigen detection tests and virus cultivation were used to detect RSV, influenza A and B viruses, parainfluenza virus types 1–3, and adenovirus. Genome-wide genotyping was performed from blood samples collected at follow-up visit at the age of 8 years.

In the VINKU2 study, nasal swabs taken at study entry were analyzed using both in-house and commercial PCR tests for RV types A, B, and C, RSV, influenza A and B viruses, parainfluenza viruses 1–3, adenovirus, coronaviruses, enteroviruses, human bocavirus, and human metapneumovirus [24, 25]. Genome-wide genotyping was performed from blood samples collected at the study entry.

In all 3 studies, genome-wide genotyping was performed on the Infinium Global Screening Array at Human Genomics Facility at Erasmus Medical Center. Eleven known asthma risk alleles in *CDHR3* gene and in the 17q21 locus were selected for analyses based on previously published studies [9–14, 16–18, 26]. These were rs6967330, rs9303277, rs3859192, rs3894194, rs2290400, rs2305480, rs11078927, rs8069176, rs7216389, rs12936231, and rs4065275.

### Definitions

In the STEPS study, ARI was defined as an episode of rhinitis or cough, with or without fever or wheezing, documented in the symptom diary by parents or diagnosed by a physician during a study clinic visit. Of all ARIs, 97.2% lasted for 30 days or less. ARIs reported to last for over 30 days were counted as separate episodes with a maximum duration of 30 days to account for overlapping infections. Children with follow-up time of at least 1 year were included in the analyses of ARIs. In the STEPS study, wheezing illnesses (bronchiolitis, recurrent wheezing, or acute exacerbation of asthma) were diagnosed by a physician based on expiratory distress, expiratory wheezing, and other signs and symptoms. Recurrent wheezing was defined as 2 or more wheezing illnesses during the follow-up time. In the VINKU and VINKU2 studies, wheezing illnesses were diagnosed by a physician and were defined by expiratory distress and distinct expiratory wheezing sound. All wheezing illnesses in the VINKU studies were considered severe because all children in VINKU and 80% in VINKU2 were hospitalized, otherwise they were treated in the emergency department of the tertiary hospital.

### Statistics

Associations between the risk alleles and the rate of ARIs were analyzed first in the STEPS birth-cohort study comprising comprehensive data on all ARIs during the first 2 years of life. Then, the associations between the risk alleles and nonwheezing and wheezing ARIs in the STEPS cohort were analyzed. Third, associations between the risk alleles and the risk of wheezing illnesses were analyzed using children in the VINKU and VINKU2 studies as cases and children in the STEPS study without wheezing illness during the follow-up as controls. Association between risk alleles and the risk of wheezing illnesses was examined by logistic regression using an additive genetic model (wild type = 0, heterozygous for risk allele = 1, homozygous for risk allele = 2). Associations between the risk alleles and the rate of ARIs (including both wheezing illnesses and nonwheezing ARIs) were analyzed in the STEPS study by negative binomial regression with natural logarithm of the follow-up time as an offset using an additive genetic model. Negative binomial model was used because of the overdispersion of the outcome data. The models were adjusted for sex and the first 5 principal components of the genetic data. The adjusted models are presented in the Supplementary Material. Two-tailed *P* values were reported, with *P* < .05 considered statistically significant. The data were analyzed using IBM SPSS Statistics software version 26 and R version 4.0.4.

## RESULTS

### Study Population and SNP Distribution

Of the 923 children in the STEPS study, genotyping was successfully done for 785 (85%) children. Of these, 748 (95%) had follow-up data on wheezing illnesses and were included in the analyses regarding wheezing illnesses (47.5% females; Table 1). Of 785 genotyped children, 694 (88%) had completed the intensive infection follow-up for at least 1 year and were included in the analyses of ARIs. A total of 247 wheezing illnesses and 7745 ARIs were documented, and 4260 nasal swabs were obtained. Of the 748 children, 128 (17%) had at least 1 wheezing illness and 53 (7%) children had recurrent (≥ 2) wheezing episodes before the age of 2 years. The incidence rate of ARIs was 6.3 (95% confidence interval [CI], 6.1–6.5) ARIs per child-year during 0–24 months of life.

**Table 1.** Baseline Characteristics of the Study Children

Characteristic	STEPS Study^a^	VINKU Studies^b^
	(n = 748)	(n = 223)
Female	355 (47.5)	67 (30.0)
Cesarean delivery	96 (12.8)	15/112 (13.4)^c^
Premature (< 37 wk)	29/744 (3.9)	9/216 (4.2)
Parental asthma	99/747 (13.3)	44 (19.7)
Parental allergy	342/729 (46.9)	111/212 (52.4)
Older sibling(s)	311 (41.6)	79/117 (67.5)^c^
Parental smoking	91/565 (16.1)	99 (44.4)
Breastfed until 6 mo of age	390/635 (61.4)	44/100 (44.0)^c^
Day care attendance^d^	167/685 (24.4)	37/117 (31.6)^c^
Eczema^d^	112/661 (16.9)	49/154 (31.8)

Data are No. in category/total No. (%).

Children in the STEPS prospective birth cohort study.

Children in the VINKU and VINKU 2 studies.

Data only from VINKU 2 study.

In the STEPS study by age 13 months; in the VINKU studies at study entry.

In the VINKU study, genotyping was successfully done for 104 (35%) of 293 wheezing children and in the VINKU2 study, for 119 (96%) of 124 wheezing children. Of 223 genotyped children, 67 (30%) were female and the median age during the hospitalization for wheezing was 13.5 (interquartile range, 7.5–19.0) months. Risk of wheezing illness was analyzed using the 223 children with the genetic data in the VINKU and VINKU2 studies as cases and 620 children in the STEPS study who did not develop wheezing illness as controls.

**Figure 1.**
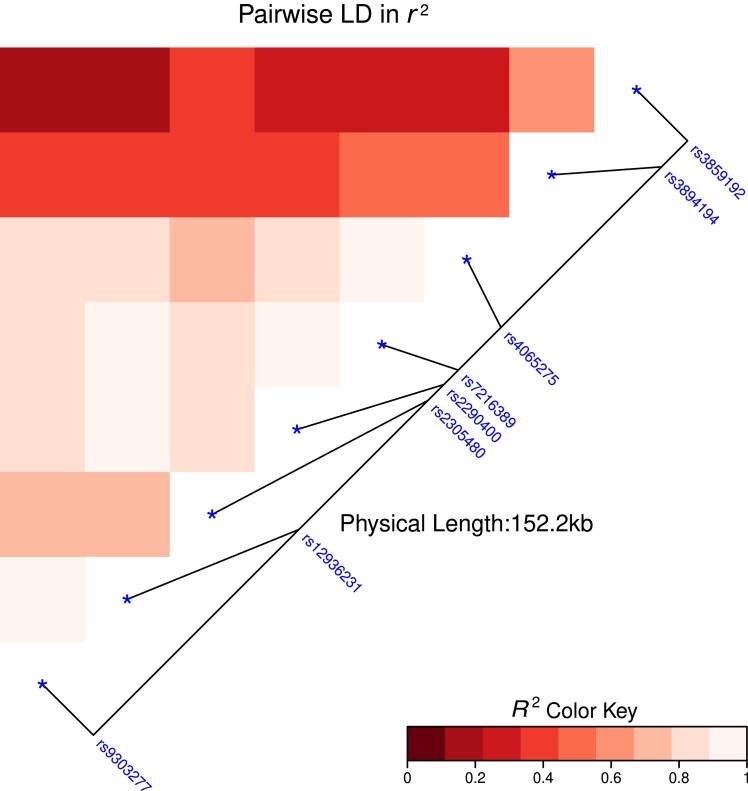
Linkage disequilibrium (LD) heatmap of locus 17q21 single-nucleotide polymorphisms (SNPs). LD heatmap of the 8 presented SNPs at 17q21 locus. Higher *r*² value denotes a stronger LD, 1 is perfect LD.

The distribution of the single-nucleotide polymorphisms (SNP) in the study children is presented in Table 2. Due to LD, 3 of the selected SNPs in *GSDMB* (rs2305480, rs11078927, and rs8069176) had almost identical distributions in the study children leading to near identical statistical findings. Hence the results are only presented for one of them (rs2305480), which is generally the best documented SNP in asthma GWAS studies. An LD heatmap of the locus 17q21 SNP's is presented in Figure 1.

**Table 2.** Genotype Distribution for Asthma Risk Alleles in the Study Children

Gene	SNP	STEPS Study^a^ (n = 748)			VINKU Studies^b^ (n = 223)		
		Number of Risk Alleles			Number of Risk Alleles		
		0	1	2	0	1	2
*CDHR3*	rs6967330-A	418 (56)	275 (37)	55 (7)	119 (53)	91 (41)	13 (6)
*IKZF3*	rs9303277-C	242 (32)	377 (50)	129 (17)	52 (23)	107 (48)	64 (29)
*GSDMA*	rs3859192-T	218 (29)	374 (50)	155 (21)	53 (24)	107 (48)	62 (28)
*GSDMA*	rs3894194-T	258 (34)	354 (47)	136 (18)	63 (28)	111 (50)	49 (22)
*GSDMB*	rs2290400-A	220 (29)	381 (51)	142 (19)	50 (22)	107 (48)	66 (30)
*GSDMB*	rs2305480-C	181 (24)	382 (51)	185 (25)	39 (18)	108 (48)	76 (34)
*GSDMB*	rs7216389-T	224 (30)	381 (51)	143 (19)	66 (30)	108 (48)	49 (22)
*ZPBP2*	rs12936231-C	234 (31)	382 (51)	132 (18)	51 (23)	107 (48)	65 (29)
*ORMDL3*	rs4065275-G	211 (28)	381 (51)	156 (21)	48 (22)	106 (48)	69 (31)

Data are No. (%).

Abbreviations: *CDHR3*, cadherin-related family member 3; *GSDMA*, gasdermin A; *GSDMB*, gasdermin B; *IKZF3*, Ikaros family zinc finger 3; *ORMDL3*, ORM1-like protein 3; SNP, single nucleotide polymorphisms; *ZPBP2*, zona pellucida binding protein 2.

Children in the STEPS prospective birth cohort study.

Children in the VINKU studies.

### Risk for Acute Respiratory Infections in the STEPS Cohort

In the STEPS study, SNPs in *GSDMA* (rs3894194-T) and *GSDMB* (rs7216389-T, rs2290400-A) were associated with a higher rate of ARI episodes while *ZPBP2* (rs12936231-C) was at the limit of statistical significance (Table 3). *GSDMA* rs3894194-T was associated with a higher rate of RV infection. *GSDMA* rs3894194-T, all studied *GSDMB* SNPs, *IKZF3* rs9303277-C, *ZPBP2* rs15936231-C, and *ORMDL3* rs4065275-G were associated with a higher risk of RSV infection. *CDHR3* SNP rs6967330 was associated with an increased rate of ARIs (incidence rate ratio [IRR], 1.06; 95% CI, 1.01–1.12; *P* = .02) and RV infections (IRR, 1.10; 95% CI, 1.01–1.20; *P* = .03). Notably, *CDHR3* rs6967330 was associated with a lower risk of RSV infection (*P* = .001).

**Table 3.** Association Between Asthma Risk Alleles and Acute Respiratory Tract Infections in the STEPS Study

Gene	SNP	ARI Episodes in the STEPS Study					
		All ARI Episodes		RV Episodes		RSV Episode	
		IRR (95% CI)	*P* Value	IRR (95% CI)	*P* Value	OR (95% CI)	*P* Value
*CDHR3*	rs6967330-A	**1.06 (1.01–1.12)**	.**02**	**1.10 (1.01–1.20)**	.**03**	**0.64 (.48–.84)**	.**001**
*IKZF3*	rs9303277-C	1.05 (1.00–1.10)	.06	1.03 (.95–1.12)	.44	**1.27 (1.00–1.61)**	.**05**
*GSDMA*	rs3859192-T	1.04 (.99–1.09)	.16	1.07 (.99–1.16)	.08	1.23 (.97–1.56)	.08
*GSDMA*	rs3894194-T	**1.05 (1.00–1.10)**	.**04**	**1.08 (1.00–1.17)**	.**05**	**1.28 (1.01–1.61)**	.**04**
*GSDMB*	rs2290400-A	**1.05 (1.00–1.10)**	.**05**	1.03 (.95–1.11)	.48	**1.35 (1.07–1.71)**	.**01**
*GSDMB*	rs2305480-C	1.05 (1.00–1.10)	.06	1.04 (.96–1.12)	.33	**1.28 (1.01–1.61)**	.**04**
*GSDMB*	rs7216389-T	**1.05 (1.00–1.11)**	.**04**	1.02 (.94–1.10)	.35	**1.31 (1.04–1.66)**	.**02**
*ZPBP2*	rs12936231-C	1.05 (1.00–1.10)	.05	1.02 (.94–1.10)	.63	**1.29 (1.01–1.63)**	.**04**
*ORMDL3*	rs4065275-G	1.04 (.99–1.09)	.14	1.01 (.94–1.09)	.76	**1.32 (1.04–1.67)**	.**02**

Values in bold are statistically significant (*P* < 0.05).Abbreviations: ARI, acute respiratory tract infection; *CDHR3*, cadherin-related family member 3; CI, confidence interval; *GSDMA*, gasdermin A; *GSDMB*, gasdermin B; *IKZF3*, Ikaros family zinc finger 3; IRR, incidence rate ratio; OR, odds ratio; *ORMDL3*, ORM1-like protein 3; RSV, respiratory syncytial virus; RV, rhinovirus; SNP, single nucleotide polymorphisms; *ZPBP2*, zona pellucida binding protein 2.

### Risk for Nonwheezing and Wheezing ARIs in the STEPS Cohort

In the STEPS study, when wheezing illnesses were excluded from the analysis, the risk allele A in *CDHR3* (rs6967330) was associated with a higher rate of all-cause nonwheezing ARIs and rhinovirus-positive ARIs, but with a smaller risk for RSV-positive ARI. Asthma risk alleles in the 17q21 locus showed a tendency of a higher rate of nonwheezing ARIs, but these findings were not statistically significant.

In the STEPS study, asthma risk alleles in *IKZF3*, *GSDMA*, *GSDMB*, *ZPBP2,* and *ORMDL3* genes were associated with a higher risk of a wheezing illness (all *P* values ≤.02; Table 4). The findings were similar for RV-positive and RSV-positive wheezing illnesses. In *CDHR3*, the rs6967330 risk allele A was not associated with wheezing illnesses in general or with RV-positive wheezing illnesses but was associated with a lower risk of RSV-positive wheezing illnesses (odds ratio [OR], 0.43; 95% CI, .22–.85; *P* = .01).

**Table 4.** Association Between Asthma Risk Alleles and Nonwheezing ARIs and Wheezing Illnesses in the STEPS Study

Gene	SNP	Nonwheezing ARI (n = 7713)						Wheezing Illness (n = 128 children)^a^					
		Any Etiology (n = 7713)		RV Positive (n = 2440)		RSV Positive (n = 191 children)^b^		Any Etiology (n = 128 children)		RV Positive (n = 46 children)		RSV Positive (n = 38 children)	
		IRR (95% CI)	*P* Value	IRR (95% CI)	*P* Value	OR (95% CI)	*P* Value	OR (95% CI)	*P* Value	OR (95% CI)	*P* Value	OR (95% CI)	*P* Value
*CDHR3*	rs6967330-A	**1.07 (1.01–1.13)**	.**02**	**1.10 (1.01–1.20)**	.**03**	**0.72 (.55–.96)**	.**03**	0.93 (.69–1.27)	.66	1.21 (.76–1.90)	.42	**0.43 (.22–.85)**	.**01**
*IKZF3*	rs9303277-C	1.04 (.99–1.09)	.16	1.02 (.94–1.10)	.66	1.13 (.89–1.45)	.32	**1.56 (1.18–2.06)**	.**002**	**2.19 (1.41–3.41)**	.**001**	**1.87 (1.16–3.02)**	.**01**
*GSDMA*	rs3859192-T	1.02 (.97–1.08)	.36	1.06 (.98–1.14)	.17	1.07 (.84–1.37)	.57	**1.46 (1.11–1.92)**	.**006**	**1.85 (1.20–2.86)**	.**006**	**1.79 (1.11–2.87)**	.**02**
*GSDMA*	rs3894194-T	1.04 (.99–1.09)	.13	1.07 (.99–1.16)	.09	1.11(.87–1.41)	.41	**1.51 (1.15–1.97)**	.**003**	**1.78 (1.17–2.71)**	.**008**	**1.96 (1.23–3.12)**	.**005**
*GSDMB*	rs2290400-A	1.04 (.99–1.09)	.13	1.01 (.94–1.10)	.75	1.20 (.94–1.53)	.15	**1.59 (1.21–2.11)**	.**001**	**2.34 (1.48–3.69)**	**<**.**001**	**1.91 (1.18–3.09)**	.**009**
*GSDMB*	rs2305480-C	1.04 (.99–1.09)	.15	1.01 (.94–1.10)	.59	1.16 (.91–1.48)	.23	**1.40 (1.06–1.85)**	.**02**	**2.45 (1.54–3.91)**	**<**.**001**	1.57 (.97–2.54)	.06
*GSDMB*	rs7216389-T	1.04 (.99–1.09)	.11	1.00 (.93–1.08)	.95	1.16 (.91–1.48)	.23	**1.57 (1.19–2.07)**	.**001**	**2.31 (1.48–3.63)**	**<**.**001**	**1.91 (1.18–3.09)**	.**008**
*ZPBP2*	rs12936231-C	1.04 (.99–1.09)	.13	1.01 (.93–1.09)	.88	1.14 (.89–1.45)	.31	**1.57 (1.19–2.07)**	.**002**	**2.25 (1.44–3.51)**	**<**.**001**	**1.94 (1.20–3.13)**	.**007**
*ORMDL3*	rs4065275-G	1.03 (.98–1.08)	.29	1.00 (.92–1.08)	.98	1.19 (.93–1.51)	.16	**1.49 (1.13–1.96)**	.**005**	**2.12 (1.36–3.32)**	.**001**	**1.76 (1.09–2.84)**	.**02**

Values in bold are statistically significant (*P* < 0.05).

Abbreviations: ARI, acute respiratory tract infection; *CDHR3*, cadherin-related family member 3; CI, confidence interval; *GSDMA*, gasdermin A; *GSDMB*, gasdermin B; *IKZF3*, Ikaros family zinc finger 3; IRR, incidence rate ratio; OR, odds ratio; *ORMDL3*, ORM1-like protein 3; RSV, respiratory syncytial virus; RV, rhinovirus; SNP, single nucleotide polymorphisms; *ZPBP2*, zona pellucida binding protein 2.^a^Of the children, 128 (17.1%) had at least one wheezing illness at age 0–24 months. In these children, a total of 247 acute wheezing illnesses were documented, of which 61 were RV-positive and 38 RSV-positive. ^b^Of the children, 191 (27.5%) had at least one non-wheezing RSV episode at age 0–24 months. In these children, 213 non-wheezing RSV-positive episodes were documented.

SNPs in *GSDMA* were associated with an increased risk of recurrent wheezing (eg, for *GSDMA* rs3859192; OR for recurrent wheezing, 1.61; 95% CI, 1.07–2.40; *P* = .02; Table 5). One SNP in *GSDMB* (rs7216389) was associated with an increased risk of recurrent wheezing while another SNP (rs2290400) was at the limit of statistical significance (*P* = .05).

**Table 5.** Association Between Asthma Risk Alleles and Recurrent Wheezing Illness in the STEPS Study

Gene	SNP	Recurrent Wheezing Illness (n = 53)	
		OR (95% CI)	*P* Value
*CDHR3*	rs6967330-A	1.09 (.71–1.69)	.70
*IKZF3*	rs9303277-C	1.40 (.94–2.10)	.10
*GSDMA*	rs3859192-T	**1.61 (1.07–2.40)**	.**02**
*GSDMA*	rs3894194-T	**1.59 (1.08–2.36)**	.**02**
*GSDMB*	rs2290400-A	1.50 (1.00–2.25)	.05
*GSDMB*	rs2305480-C	1.44 (.96–2.16)	.08
*GSDMB*	rs7216389-T	**1.51 (1.01–2.26)**	.**05**
*ZPBP2*	rs12936231-C	1.42 (.95–2.13)	.09
*ORMDL3*	rs4065275-G	1.39 (.93–2.08)	.11

Values in bold are statistically significant (*P* < 0.05).Abbreviations: *CDHR3*, cadherin-related family member 3; CI, confidence interval; *GSDMA*, gasdermin A; *GSDMB*, gasdermin B; *IKZF3*, Ikaros family zinc finger 3; OR, odds ratio; *ORMDL3*, ORM1-like protein 3; SNP, single nucleotide polymorphisms; *ZPBP2*, zona pellucida binding protein 2.

### Risk for Severe Wheezing Illness in the VINKU Studies

In the combined VINKU and VINKU2 data investigating severe wheezing illnesses, the findings were mainly similar. Risk alleles in *IKZF3*, *GSDMA*, *GSDMB*, *ZPBP2,* and *ORMDL3* were associated with an increased risk of a severe wheezing illness (Table 6). In line with the STEPS study, risk alleles in *IKZF3*, *GSDMA*, *GSDMB*, *ZPBP2,* and *ORMDL3* were associated with an increased risk of RV-positive severe wheezing illnesses. However, unlike in the STEPS study, these alleles were not associated with the risk of RSV-positive wheezing illness. In the combined VINKU and VINKU2 data, the *CDHR3* SNP rs6967330 was not associated with severe wheezing illnesses.

**Table 6.** Association Between Asthma Risk Alleles and Severe Wheezing Illnesses in the VINKU Studies (n = 843 With Controls From STEPS Study)^a^

Gene	SNP	Severe Wheezing Illness					
		Any Etiology (n = 223)		RV Positive (n = 129)		RSV Positive (n = 63)	
		OR (95% CI)	*P* Value	OR (95% CI)	*P* Value	OR (95% CI)	*P* Value
*CDHR3*	rs6967330-A	1.01 (.79–1.29)	.91	1.17 (.87–1.57)	.30	0.80 (.52–1.23)	.32
*IKZF3*	rs9303277-C	**1.65 (1.32–2.06)**	**<**.**001**	**1.66 (1.27–2.18)**	**<**.**001**	1.23 (.85–1.77)	.28
*GSDMA*	rs3859192-T	**1.37 (1.10–1.71)**	.**005**	**1.50 (1.14–1.96)**	.**003**	1.06 (.74–1.53)	.75
*GSDMA*	rs3894194-T	**1.31 (1.06–1.63)**	.**01**	**1.58 (1.21–2.07)**	.**001**	0.97 (.68–1.40)	.89
*GSDMB*	rs2290400-A	**1.56 (1.25–1.96)**	**<**.**001**	**1.64 (1.25–2.16)**	**<**.**001**	1.17 (.81–1.69)	.41
*GSDMB*	rs2305480-C	**1.48 (1.18–1.85)**	.**001**	**1.48 (1.12–1.95)**	.**005**	1.08 (.75–1.57)	.67
*GSDMB*	rs7216389-T	**1.59 (1.27–1.98)**	**<**.**001**	**1.68 (1.27–2.20)**	**<**.**001**	1.17 (.81–1.69)	.40
*ZPBP2*	rs12936231-C	**1.64 (1.31–2.45)**	**<**.**001**	**1.68 (1.28–2.21)**	**<**.**001**	1.19 (.83–1.72)	.35
*ORMDL3*	rs4065275-G	**1.51 (1.21–1.88)**	**<**.**001**	**1.62 (1.24–2.13)**	**<**.**001**	1.16 (.81–1.68)	.42

Values in bold are statistically significant (*P* < 0.05).Abbreviations: *CDHR3*, cadherin-related family member 3; CI, confidence interval; *GSDMA*, gasdermin A; *GSDMB*, gasdermin B; *IKZF3*, Ikaros family zinc finger 3; OR, odds ratio; *ORMDL3*, ORM1-like protein 3; RSV, respiratory syncytial virus; RV, rhinovirus; SNP, single nucleotide polymorphisms; *ZPBP2*, zona pellucida binding protein 2.

Children in the VINKU studies with severe wheezing illness treated at hospital (n = 223) were used as cases and STEPS study children without wheezing (n = 620) as controls.

## DISCUSSION

In this study, we found, first, that previously identified asthma risk alleles in *CDHR3*, *GSDMA,* and *GSDMB* were associated with an increased rate of all ARIs, risk alleles in *CDHR3* and *GSDMA* with an increased rate of RV-positive ARIs, and risk alleles in *GSDMA, GSDMB, IKZF3, ZPBP2,* and *ORMDL3* with an increased risk of RSV-positive ARI. Second, when wheezing and nonwheezing ARIs were analyzed separately, alleles at the 17q21 locus genes (*GSDMA*, *GSDMB*, *IKZF3*, *ZPBP2,* and *ORMDL3*) were associated with an increased risk of all wheezing illnesses and RV-positive wheezing illnesses in the early childhood but not significantly with nonwheezing ARIs. Third, contrary to the association with RV infections, the risk allele in *CDHR3* was associated with a lower risk of RSV-positive ARIs.

Previous studies have reported that the 17q21 locus alleles in genes *GSDMA*, *GSDMB*, *IKZF3, ZPBP2,* and *ORMDL3* are associated with increased risk and severity of childhood asthma and increased number of early wheezing illnesses [6–12]. Of these genes, *GSDMB*, *IKZF3*, *ZPBP2,* and *ORMDL3* are in LD while *GSDMA* is situated in a more distal region of the 17q21 locus and shows less LD with the other genes in Europeans [7]. Mechanisms behind these genetic associations are poorly known, and the functions of the genes are only partially known [14]. Gasdermin B encoded by *GSDMB* mediates epithelial cell pyroptosis [13]. *ORMDL3* gene encodes ORMDL3 protein, which is an inhibitor of sphingolipid synthesis [7, 8]. The asthma risk alleles in *ORMDL3* affect the expression of *ORMDL3* and have subsequent effects in binding of the protein and interleukin production in blood [26] and nasal epithelial cells, where it was recently shown that sphingolipid levels depend on the *ORMDL3* expression levels [27]. *IKZF* encodes Ikaros family zinc finger protein 3 involved in lymphocyte differentiation [28]. *ZPBP2* encodes zona pellucida binding protein 2 and SNP rs12936231 in *ZPBP2* affects ORMDL3 expression [6].

We found that all studied risk alleles at the 17q21 locus were associated with an increased risk of wheezing illness both in the STEPS birth cohort and in combined VINKU data. Furthermore, risk alleles in *GSDMA* and *GSDMB* were significantly associated with recurrent wheezing illnesses in the STEPS birth cohort, and risk alleles in other 17q21 locus genes had weaker, nonsignificant associations. Corresponding findings both from an unselected birth cohort (STEPS) and from children with severe wheezing illness treated at hospitals (VINKU) strengthen the perception of similar pathobiology of early childhood wheezing illnesses and later asthma. However, there were differences between the STEPS and VINKU study findings regarding genetic risk for virus-specific wheezing illnesses. In the STEPS study, all the 17q21 locus risk alleles were associated with RV- and RSV-positive wheezing illness, whereas in the VINKU studies associations were significant only for RV-positive wheezing, and associations for RSV-positive wheezing illnesses were weak and nonsignificant. In part, these findings may be related to the low number of RSV-positive wheezing children in the VINKU studies but differences in the study populations should be noted. Almost all wheezing illnesses in the STEPS birth-cohort study were mild and children were managed as outpatients, as reported earlier [21, 29], whereas VINKU studies recruited only children with severe wheezing illness treated at hospital. Earlier birth-cohort studies reporting association of the 17q21 locus alleles with RV-positive but not with RSV-positive wheezing illnesses have included only children who had parent(s) with asthma or respiratory allergies [17].

*CDHR3* gene encodes CDHR3 transmembrane protein, which is mainly expressed in the epithelium of the airways and is the only identified receptor for RV types belonging to species C [15]. Previous studies have shown that allele A at rs6967330 in *CDHR3* is associated with an increased risk and increased severity of childhood asthma [9]. In our study, it was not significantly associated with an increased risk for wheezing illness, RV-positive wheezing illness, or recurrent wheezing. In particular, RV-positive wheezing illness and recurrent wheezing are strong risk factors for later asthma. Our findings suggest that the CDHR3 variant-related genetic risk for early life RV-induced wheezing and later RV-related asthma pathology may differ from each other, or alternatively, our study may have been underpowered to detect weak associations with these outcomes.

Earlier studies conducted in children with increased risk for asthma have shown that allele A at *CDHR3* rs6967330 is associated with an increased risk for ARIs, specifically for ARIs caused by RV species C [30]. Our findings add to the previous literature by demonstrating in a large, unselected birth-cohort that this *CDHR3* allele is associated with an increased rate of all ARIs and RV-positive ARIs. This is biologically plausible as allele A at *CDHR3* rs6967330, has been shown to facilitate receptor binding and replication of RV species C in transfected cells [15].

Interestingly, the asthma risk allele in *CDHR3* was associated with a reduced risk of RSV-positive wheezing illness and RSV-positive ARIs in the STEPS birth cohort. In VINKU studies involving children with severe wheezing illness, similar association was not confirmed or ruled out (OR, 0.80, CI .52–1.23). As CDHR3 is not known to have a role in the pathogenesis of RSV infection, the mechanism behind this association remains unknown. Viral interference between RV and RSV has been demonstrated [31] and could be involved in this phenomenon.

In addition to *CDHR3* rs6967330, asthma risk alleles at the 17q21 locus seemed to be associated with an increased rate of all ARIs in the STEPS birth cohort. The associations were rather weak and significant only for *GSDMA* (rs3894194) and *GSDMB* (rs2290400 and rs7216389). Interestingly, there were no clear associations between 17q21 locus alleles and RV infections, with only *GSDMA* (rs3894194) being statistically significant. RV is the most frequent causative agent of ARIs in children and thus dominates the findings for all ARIs. In contrast, we found most asthma risk alleles at the 17q21 locus to have significant association with increased rate of RSV infections. Most RSV infections in the STEPS study children manifested as mild upper or lower respiratory illnesses, and only 2% needed hospitalization [29]. Earlier studies have assessed the genetic risk for severe hospital-treated RSV bronchiolitis and found associations with variant forms of innate immune and other genes [32]. Considering earlier literature in light of our present findings, it seems that genetic risk factors have different roles in the susceptibility to mild and severe manifestations of RSV infection.

Our study has potential limitations. First, although we analyzed data from a large birth-cohort study and from 2 large prospective studies in children with severe wheezing, the groups of outpatients or hospitalized patients with wheezing caused by specific viruses were of limited size, decreasing the power to detect weak genetic associations. Second, we did not have information on species distribution of RVs. Third, due to the large number of comparisons, there is a possibility of coincidental findings. Fourth, this study was conducted in Finnish children, which is to be noted when generalizing the results to other study populations.

## CONCLUSION

In this study, known asthma risk alleles were associated with an increased rate of ARIs and an increased risk of viral, especially RV-induced, wheezing illnesses in early childhood. This suggests that susceptibility to recurrent ARIs in early childhood may share genetic risk factors with wheezing illnesses and asthma. The asthma risk allele in *CDHR3*, coding for RV species C receptor, was associated with an increased rate of RV infections and a decreased risk of RSV infection. Thus, a genetic variant can have opposite effects on risks of specific respiratory virus infections.

## Supplementary Data

Supplementary materials are available at *The Journal of Infectious Diseases* online. Consisting of data provided by the authors to benefit the reader, the posted materials are not copyedited and are the sole responsibility of the authors, so questions or comments should be addressed to the corresponding author.

## Supplementary Material

jiad075_Supplementary_Data

## Notes

***Acknowledgments*.** We thank all the families who participated in this study, the midwives for their help in recruiting the families, and the whole STEPS study team for assistance with data collection, Hanna Lagström, PhD, for her many contributions to the STEPS study, and study nurses for their assistance in the study clinic. We also thank Ari Ahola-Olli, MD, PhD, at Turku University Hospital and University of Turku, for his help with the illustrations.

***Financial support*.** This work was supported by the University of Turku; the Abo Akademi University; the Turku University Hospital; the Academy of Finland (grants numbers 123571, 140251, 277535, and 324926); the Emil Aaltonen Foundation; the Sigrid Juselius Foundation; the Finnish Medical Foundation; the Päivikki and Sakari Sohlberg Foundation; the Foundation for Pediatric Research; the Allergy Research Foundation; the Paulo Foundation; research funds from specified government transfers, Hospital District of Southwest Finland; the Tampere Tuberculosis Foundation; and the Juho Vainio Foundation.
