# Supplementary material for: Association of Asthma Risk Alleles With Acute Respiratory Tract Infections and Wheezing Illnesses in Young Children
Source: J Infect Dis. 2023 Mar 27;228(8):990–8. doi: 10.1093/infdis/jiad075 (PMC10582910; doi:10.1093/infdis/jiad075)
Supplement: jiad075_Supplementary_Data [file jiad075_supplementary_data.docx]

**Supplementary material**

**Association of Asthma Risk Alleles With Acute Respiratory Tract Infections and Wheezing Illnesses in Young Children**

Ville Forsström, MD^1^, Laura Toivonen, MD, PhD^1^, Kiara Homil, BM^1^, Matti Waris, PhD^2^, Casper-Emil T. Pedersen, PhD^3^, Klaus Bønnelykke, MD, PhD^3^, Tuomas Jartti, MD, PhD^1,4,5^, Ville Peltola, MD, PhD^1^.

^1^ Department of Paediatrics and Adolescent Medicine, Turku University Hospital and University of Turku, Turku, Finland

^2^ Virology Unit, Institute of Biomedicine, University of Turku, Turku, Finland

^3^ Copenhagen Prospective Studies on Asthma in Childhood, Herlev and Gentofte Hospital, University of Copenhagen, Copenhagen, Denmark

^4^PEDEGO Research Unit, University of Oulu, Oulu, Finland

^5^Department of Pediatrics and Adolescent Medicine, University of Oulu, Oulu, Finland

**Supplementary Results**

**Supplementary tables adjusted for sex and first five principal components of the genetic data.**

**Supplementary table 1.** **Association between asthma risk alleles and acute respiratory tract infections (ARI) in the STEPS Study, adjusted for sex and first five principal components of the genetic data.**

|  |  | ARI episodes in the STEPS Study (n = 7957) | | | | | |
| --- | --- | --- | --- | --- | --- | --- | --- |
| **Gene** | **SNP** | **All ARI episodes (n = 7957)** | | **RV episodes (n = 2500)** | | **RSV episode (n = 220 children)^1^** | |
|  |  | IRR (95% CI) | P value | IRR (95% CI) | P value | OR (95% CI) | P value |
| *CDHR3* | rs6967330-A | **1.07 (1.01-1.13)** | **0.02** | **1.10 (1.01-1.20)** | **0.03** | **0.63 (0.48-0.83)** | **0.001** |
| *IKZF3* | rs9303277-C | 1.05 (1.00-1.10) | 0.08 | 1.03 (0.95-1.11) | 0.48 | **1.27 (1.00-1.61)** | **0.05** |
| *GSDMA* | rs3859192-T | 1.04 (0.99-1.09) | 0.15 | 1.07 (0.99-1.16) | 0.08 | 1.24 (0.98-1.57) | 0.08 |
| *GSDMA* | rs3894194-T | **1.05 (1.00-1.10)** | **0.04** | **1.08 (1.00-1.17)** | **0.05** | **1.29 (1.02-1.63)** | **0.03** |
| *GSDMB* | rs2290400-A | **1.05 (1.00-1.10)** | **0.05** | 1.03 (0.95-1.11) | 0.50 | **1.35 (1.07-1.72)** | **0.01** |
| *GSDMB* | rs2305480-C | 1.05 (1.00-1.10) | 0.05 | 1.04 (0.97-1.13) | 0.28 | **1.29 (1.01-1.63)** | **0.04** |
| *GSDMB* | rs7216389-T | **1.05 (1.00-1.11)** | **0.04** | 1.02 (0.95-1.11) | 0.56 | **1.33 (1.05-1.68)** | **0.02** |
| *ZPBP2* | rs12936231-C | 1.05 (1.00-1.10) | 0.06 | 1.02 (0.94-1.10) | 0.66 | **1.28 (1.01-1.62)** | **0.04** |
| *ORMDL3* | rs4065275-G | 1.04 (0.99-1.09) | 0.14 | 1.01 (0.94-1.10) | 0.73 | **1.33 (1.05-1.68)** | **0.02** |

Abbreviations: *CDHR3*, cadherin-related family member 3; CI, confidence interval; *GSDMA*, gasdermin A; *GSDMB*, gasdermin B; *IKZF3*, ikaros family zinc finger 3; IRR, incidence rate ratio; OR, odds ratio; *ORMDL3*, ORM1-like protein 3; RSV, respiratory syncytial virus; RV, rhinovirus; SNP, single nucleotide polymorphisms; *ZPBP2*, zona pellucida binding protein 2

^1^ RSV-positive ARI was documented in 220 (33.5%) children (a total of 250 RSV-positive ARIs).

**Supplementary table 2.** **Association between asthma risk alleles and non-wheezing ARIs and wheezing illnesses in the STEPS Study, adjusted for sex and first five principal components of the genetic data.**

|  |  | STEPS Study (n = 748 children) | | | | | | | | | | | |
| --- | --- | --- | --- | --- | --- | --- | --- | --- | --- | --- | --- | --- | --- |
| **Gene** | **SNP** | **Non-Wheezing ARIs (n = 7713)** | | | | | | **Wheezing illness (n = 128 children)^1^** | | | | | |
|  |  | Any aetiology (n = 7713) | | RV-positive (n = 2440) | | RSV-positive (n = 191 children)^2^ | | Any aetiology (n = 128 children) | | RV-positive (n = 46 children) | | RSV-positive (n = 38 children) | |
|  |  | IRR (95% CI) | P value | IRR (95% CI) | P value | OR (95% CI) | P value | OR (95% CI) | P value | OR (95% CI) | P value | OR (95% CI) | P value |
| *CDHR3* | rs6967330-A | **1.07 (1.01-1.13)** | **0.01** | **1.10 (1.01-1.20)** | **0.03** | **0.70 (0.53-0.92)** | **0.012** | 0.95 (0.70-1.30) | 0.76 | 1.25 (0.79-1.99) | 0.35 | **0.45 (0.23-0.87)** | **0.02** |
| *IKZF3* | rs9303277-C | 1.03 (0.98-1.09) | 0.19 | 1.02 (0.94-1.10) | 0.70 | 1.09 (0.86-1.39) | 0.47 | **1.57 (1.19-2.08)** | **0.002** | **2.33 (1.47-3.69)** | **<0.001** | **1.87 (1.15-3.02)** | **0.01** |
| *GSDMA* | rs3859192-T | 1.02 (0.97-1.08) | 0.34 | 1.06 (0.98-1.15) | 0.16 | 1.01 (0.80-1.28) | 0.93 | **1.52 (1.15-2.01)** | **0.003** | **2.05 (1.29-3.25)** | **0.002** | **1.86 (1.15-3.02)** | **0.004** |
| *GSDMA* | rs3894194-T | 1.04 (0.99-1.09) | 0.12 | 1.07 (0.99-1.16) | 0.08 | 1.07 (0.85-1.35) | 0.57 | **1.54 (1.17-2.03)** | **0.002** | **1.91 (1.23-2.99)** | **0.004** | **2.00 (1.25-3.20)** | **0.004** |
| *GSDMB* | rs2290400-A | 1.04 (0.99-1.09) | 0.14 | 1.01 (0.94-1.10) | 0.75 | 1.15 (0.90-1.46) | 0.26 | **1.62 (1.22-2.15)** | **0.001** | **2.52 (1.57-4.06)** | **<0.001** | **1.92 (1.18-3.12)** | **0.009** |
| *GSDMB* | rs2305480-C | 1.04 (0.99-1.09) | 0.13 | 1.03 (0.95-1.11) | 0.52 | 1.11 (0.88-1.41) | 0.37 | **1.45 (1.10-1.93)** | **0.01** | **2.87 (1.74-4.73)** | **<0.001** | 1.60 (0.99-2.60) | 0.06 |
| *GSDMB* | rs7216389-T | 1.04 (0.99-1.09) | 0.11 | 1.01 (0.93-1.09) | 0.81 | 1.11 (0.88-1.41) | 0.38 | **1.59 (1.20-2.11)** | **0.001** | **2.47 (1.55-3.94)** | **<0.001** | **1.91 (1.18-3.10)** | **0.009** |
| *ZPBP2* | rs12936231-C | 1.04 (0.99-1.09) | 0.15 | 1.01 (0.93-1.09) | 0.90 | 1.09 (0.86-1.39) | 0.46 | **1.58 (1.19-2.10)** | **0.001** | **2.35 (1.49-3.73)** | **<0.001** | **1.94 (1.19-3.15)** | **0.007** |
| *ORMDL3* | rs4065275-G | 1.03 (0.98-1.08) | 0.29 | 1.00 (0.93-1.08) | 0.97 | 1.14 (0.90-1.45) | 0.27 | **1.52 (1.15-2.02)** | **0.003** | **2.31 (1.45-3.68)** | **<0.001** | **1.78 (1.10-2.89)** | **0.02** |

Abbreviations: *CDHR3*, cadherin-related family member 3; CI, confidence interval; *GSDMA*, gasdermin A; *GSDMB*, gasdermin B; *IKZF3*, ikaros family zinc finger 3; OR, odds ratio; *ORMDL3*, ORM1-like protein 3; RSV, respiratory syncytial virus; RV, rhinovirus; SNP, single nucleotide polymorphisms; *ZPBP2*, zona pellucida binding protein 2.

^1^ Of the children, 128 (17.1%) had at least one wheezing illness at age 0-24 months. In these children, a total of 247 acute wheezing illnesses were documented, of which 61 were RV-positive and 38 RSV-positive.

^2^ Of the children, 191 (27.5%) had at least one non-wheezing RSV episode at age 0-24 months. In these children, 213 non-wheezing RSV-positive episodes were documented.

**Supplementary table 3.** **Association between asthma risk alleles and recurrent wheezing illness in the STEPS Study, adjusted for sex and first five principal components of the genetic data.**

| **Gene** | **SNP** | **Recurrent wheezing illness (n = 53 children)** | |
| --- | --- | --- | --- |
|  |  | OR (95% CI) | P value |
| *CDHR3* | rs6967330-A | 1.11 (0.72-1.72) | 0.63 |
| *IKZF3* | rs9303277-C | 1.43 (0.95-2.14) | 0.09 |
| *GSDMA* | rs3859192-T | **1.69 (1.12-2.57)** | **0.01** |
| *GSDMA* | rs3894194-T | **1.67 (1.11-2.50)** | **0.01** |
| *GSDMB* | rs2290400-A | 1.52 (1.01-2.30) | 0.04 |
| *GSDMB* | rs2305480-C | 1.52 (1.01-2.28) | 0.06 |
| *GSDMB* | rs7216389-T | **1.53 (1.02-2.30)** | **0.04** |
| *ZPBP2* | rs12936231-C | 1.44 (0.96-2.15) | 0.08 |
| *ORMDL3* | rs4065275-G | 1.43 (0.95-2.15) | 0.09 |

Abbreviations: *CDHR3*, cadherin-related family member 3; CI, confidence interval; *GSDMA*, gasdermin A; *GSDMB*, gasdermin B; *IKZF3*, ikaros family zinc finger 3; OR, odds ratio; *ORMDL3*, ORM1-like protein 3; SNP, single nucleotide polymorphisms; *ZPBP2*, zona pellucida binding protein 2.

**Supplementary table 4. Association between asthma risk alleles and wheezing illnesses in the VINKU Studies, adjusted for sex and first five principal components of the genetic data.**

|  |  | VINKU Studies^1^ (n = 843 children with controls from STEPS Study) | | | | | |
| --- | --- | --- | --- | --- | --- | --- | --- |
| **Gene** | **SNP** | **Severe wheezing illness (n = 223 children)** | | | | | |
|  |  | Any aetiology (n = 223 children) | | RV-positive (n = 129 children) | | RSV-positive (n = 63 children) | |
|  |  | OR (95% CI) | P value | OR (95% CI) | P value | OR (95% CI) | P value |
| *CDHR3* | rs6967330-A | 1.02 (0.80-1.31) | 0.85 | 1.17 (0.87-1.57) | 0.31 | 0.79 (0.51-1.22) | 0.29 |
| *IKZF3* | rs9303277-C | **1.69 (1.34-2.12)** | **<0.001** | **1.67 (1.27-2.21)** | **<0.001** | 1.25 (0.87-1.81) | 0.23 |
| *GSDMA* | rs3859192-T | **1.49 (1.18-1.87)** | **0.001** | **1.59 (1.21-2.09)** | **0.001** | 1.10 (0.76-1.58) | 0.62 |
| *GSDMA* | rs3894194-T | **1.36 (1.09-1.70)** | **0.007** | **1.62 (1.24-2.13)** | **0.001** | 0.98 (0.68-1.42) | 0.92 |
| *GSDMB* | rs2290400-A | **1.62 (1.28-2.04)** | **<0.001** | **1.66 (1.26-2.20)** | **<0.001** | 1.19 (0.82-1.73) | 0.36 |
| *GSDMB* | rs2305480-C | **1.56 (1.24-1.97)** | **<0.001** | **1.52 (1.14-2.01)** | **0.004** | 1.12 (0.77-1.64) | 0.54 |
| *GSDMB* | rs7216389-T | **1.62 (1.29-2.04)** | **<0.001** | **1.68 (1.27-2.23)** | **<0.001** | 1.19 (0.82-1.72) | 0.36 |
| *ZPBP2* | rs12936231-C | **1.68 (1.33-2.11)** | **<0.001** | **1.69 (1.28-2.24)** | **<0.001** | 1.22 (0.84-1.76) | 0.30 |
| *ORMDL3* | rs4065275-G | **1.56 (1.24-1.95)** | **<0.001** | **1.65 (1.25-2.17)** | **<0.001** | 1.18 (0.82-1.71) | 0.37 |

Abbreviations: *CDHR3*, cadherin-related family member 3; CI, confidence interval; *GSDMA*, gasdermin A; *GSDMB*, gasdermin B; *IKZF3*, ikaros family zinc finger 3; OR, odds ratio; *ORMDL3*, ORM1-like protein 3; RSV, respiratory syncytial virus; RV, rhinovirus; SNP, single nucleotide polymorphisms; *ZPBP2*, zona pellucida binding protein 2.

^1^ Children in the VINKU studies with severe wheezing treated at hospital (n = 223) were used as cases and STEPS Study children without wheezing (n = 620) as controls.
